# Uptake and survival of *Leishmania **amazonensis*** in *Acanthamoeba*: an adequate model organism?

**DOI:** 10.1590/0074-02760250253

**Published:** 2026-06-19

**Authors:** Leonardo Fernandes Geres, Pedro Henrique Gallo Francisco, Diullia de Andrade Machado, Francisco Breno Silva Teófilo, Marcelo Brocchi, Selma Giorgio

**Affiliations:** 1 Universidade Estadual de Campinas, Instituto de Biologia, Departamento de Biologia Animal, Campinas, SP, Brasil; 2 Universidade Estadual de Campinas, Instituto de Biologia, Departamento de Biologia Molecular e Morfofuncional, Campinas, SP, Brasil; 3 Universidade Estadual de Campinas, Instituto de Biologia, Departamento de Genética, Evolução, Microbiologia e Imunologia, Campinas, SP, Brasil

**Keywords:** Acanthamoeba, Leishmania, models, protozoa

## Abstract

**BACKGROUND:**

Research on *Leishmania* pathogenesis, as well as drug and vaccine discovery, primarily relies on complex mammalian models that raise ethical concerns. To overcome these limitations, we tested a simpler and more accessible experimental system. In this study, we examined the interaction between *Leishmania **amazonensis*** and *Acanthamoeba*, a widespread free-living protozoan that interacts with various microorganisms.

**OBJECTIVES:**

To provide a deeper morphological and kinetic characterisation of the interaction between *L. **amazonensis*** and *Acanthamoeba*.

**METHODS:**

The parasite interaction was characterised using light microscopy, fluorescence microscopy, scanning electron microscopy (SEM), confocal microscopy, and live-cell imaging.

**FINDINGS:**

Co-culture was most optimal in RPMI medium at 26ºC. *L. **amazonensis*** promastigotes invaded *Acanthamoeba* trophozoites via their flagellum; trophozoites phagocytosed parasites through acanthopodia. Inside the amoeba, *L. **amazonensis*** became rounded and shortened, with no visible flagellum. These forms, isolated after 3 h of co-culture, differentiated back into promastigotes and were viable. The percentage of amoebas with *L. **amazonensis*** decreased over time.

**MAIN CONCLUSIONS:**

*Acanthamoeba* trophozoites can interact and clear out *L. **amazonensis***. This model is unsuited for sustained infection studies necessary for drug screening. However, it could be an effective model for exploring cellular leishmanicidal mechanisms.

Leishmaniasis is classified by the World Health Organisation (WHO) as a neglected tropical disease.^1^ This parasitic disease is a global public health concern associated with impoverished populations.^2^ It is estimated that approximately 350 million people are at risk of infection, with about 1 million new cases reported annually.^1^ Epidemiological data indicate that the incidence of this disease has increased significantly in urban areas.^1^

*Leishmania* spp. is transmitted through the blood meal of infected female sandflies from the genera *Phlebotomus* (Africa, Europe, and Asia) and *Lutzomyia* (mainly in South and Central America).^1^ After replicating in the sandfly's digestive tract, promastigotes differentiate into the infective form, the metacyclic promastigotes. These are regurgitated during the blood meal, along with the insect's saliva, into the dermis of the mammalian host.^3^ The inoculated saliva attracts phagocytic cells to the bite site; once phagocytosed by the host's cells of the mononuclear phagocyte system, the promastigotes transform into amastigotes and start to proliferate.^3^

Among the various clinical manifestations of the disease, cutaneous leishmaniasis is the most common. This form typically occurs on exposed skin areas, with an erythematous base and well-defined edges.^4^ Mucocutaneous leishmaniasis damages mucosal tissue, mainly in the nasopharyngeal region, while visceral leishmaniasis is the most severe form and can be fatal if not treated.^1,2^ There are a few drugs available, and they often cause serious side effects and high treatment costs.^2^ Additionally, there are currently no vaccines to prevent leishmaniasis in humans. To develop more effective treatments, it is crucial to establish suitable models that can replicate the cellular environment and the parasite's life cycle.

Model organisms must necessarily represent the desired system and be easier to study than the target being modelled.^5^ *Leishmania* spp. strategies of infection, microbicidal functions, immunomodulation, and drug effectiveness have been inferred mainly from studies conducted in murine models, primary macrophages, and immortalised cell lines, and, more recently, from studies using three-dimensional cell culture models.^6^,^7^,^8^ However, all these models have limitations, and no single model mimics all aspects of infection and pathogenesis. It is expected that we will have different models that yield different answers, given that parasite development in humans is highly uncertain and depends on several factors, including virulence, immunological factors, the host microbiota, and *Leishmania* species.^9^ Therefore, developing additional models can help in understanding the diversity of the *Leishmania*-host relationship.

There is a wide diversity of model systems in protozoa, including *Acanthamoeba*, which are characterised by short generation times and easy storage, handling, and identification (morphological, genetic, and biochemical data), as well as phenotypic stability.^10^ *Acanthamoeba* is a ubiquitous, free-living protozoan that reproduces by mitosis, and its trophozoites are associated with biofilms.^11^,^12^ Cysts can be transmitted through the air (wind and dust storms) and via long-distance ventilation ducts.^13^

*Acanthamoeba* is known to interact with various microorganisms, including bacteria (*e.g.*, *Legionella pneumophila*), fungi (*e.g.*, *Cryptococcus* spp.), viruses (*e.g.*, Yaravirus), and protozoa (*e.g.*, *Toxoplasma gondii* and *Cryptosporidium parvum*), making it a potential model organism for studying *Leishmania* spp. Infection.^14^,^15^,^16^,^17^,^18^,^19^,^20^,^21^ These amoebas may act as a transmission vehicle when the microorganism lives within the amoeba without multiplying, or as a reservoir when the microorganism proliferates within the host.^16^ In addition, *Acanthamoeba* and mammalian macrophages share similarities in structure, cellular physiology, the presence of digestive vacuoles, phagotrophic capacity, and the ability to eliminate microorganisms.^16^,^17^,^22^ For example, oxidative attack in amoeba is similar to that observed in macrophages in the presence of reactive oxygen species (ROS) and nitric oxide (NO).^16^,^17^ The hypothesis that *Acanthamoeba*, at some point in its evolutionary history, may have been a host for *Leishmania* spp. and "trained" them to evade phagocytes and, consequently, the innate immune system, has previously been suggested, given that the two protists have been present on Earth for millions of years.^23^

There are only a few reports on the interaction between *Leishmania* spp. and amoeba, and the details of their relationship remain poorly understood.^18,24,25^ Based on electron microscopy image analysis, it has been suggested that the interaction between *Acanthamoeba* and *Leishmania* spp. results in the destruction of the amoeba,^24^ while another study analysing optical microscopy images concluded that *Leishmania* spp. subvert amoeba functions.^25^ Therefore, the main goal of this study was to provide a detailed morphological and kinetic characterisation of the interaction between these two protozoans.

## MATERIALS AND METHODS

*Parasite culture* - Promastigotes of *L. **amazonensis*** (MHOM/BR/73/M2269), specie easily cultivated *in vitro* and highly infective in macrophages and murine models,^26^ were maintained in RPMI-1640 medium (Sinergia, Campinas, Brazil) at pH 7.4 supplemented with 10% foetal bovine serum (FBS) (Vitrocell, Freiburg, Germany) and 50 µg/mL gentamicin (Sigma-Aldrich. St. Louis, MO, USA) and grown in 25 cm^2^ cell culture flasks at 26ºC. The promastigote forms of *L. **amazonensis*** expressing green fluorescent protein [*L. **amazonensis***-green fluorescent protein (GFP)] (strain MHOM/BR/75/Josefa) were maintained in RPMI culture medium + 10% FBS at pH 7.4, periodically selected with 200 µg/mL geneticin (G418) (Sigma-Aldrich), and grown in 25 cm^2^ cell culture flasks at 26ºC.^26^,^27^

*Acanthamoeba castellanii* trophozoites (ATCC 30010) were kindly provided by Dr. Cristina Elisa Alvez Martinez from the Department of Genetics, Evolution, Microbiology, and Immunology at the Institute of Biology, Universidade Estadual de Campinas (UNICAMP). The strain ATCC 30010 has been historically classified as *A. **castellanii***. However, a recent taxonomic revision has reclassified it as *A. **terricola***.^28^ To ensure taxonomic accuracy while maintaining clarity with the historical literature throughout the manuscript, we used only the genus name *Acanthamoeba*. Trophozoites were maintained in axenic peptone-yeast extract medium + 0.1 M glucose (PYG) at pH 6.5 supplemented with 300 µg/mL streptomycin and 100 µg/mL ampicillin, and grown in 25 cm^2^ cell culture flasks at 26ºC.^18^

*Parasite proliferation curves* - Promastigotes or trophozoites cultures were quantified, and 5 × 10^5^ parasites/mL were cultured in 6-well plates at a final volume of 5 mL in the indicated medium (RPMI, PYG, and PYG + 10% FBS) at 26ºC, 34ºC, or 37ºC. At 24 h intervals, parasites were quantified using a Neubauer chamber for 10 days. The assay was performed in biological triplicate for each proliferation curve produced.

*Uptake assays* - Assays were performed in 24-well plates with 13-mm-diameter coverslips. The interaction between *Acanthamoeba* trophozoites and *L. **amazonensis*** promastigotes or fluorescein isothiocyanate (FITC) labelled microbeads (diameter 2 μm) (Thermo Fisher, MA, USA) was carried out in PYG medium + 10% FBS or in RPMI medium + 10% FBS. The plates were incubated at 26ºC or 34ºC. 3 × 10^5^ trophozoites were added to the wells at ratios of 1:10 and 1:20 (trophozoite: promastigotes). To achieve the stated ratios: 3 × 10^6^ promastigotes were added for the 1:10 ratio (trophozoite: promastigotes); For the 1:20 ratio, 6 × 10^6^ promastigotes were added. Microbeads were added to the wells at a ratio of 1:10 (trophozoite: microbead). The coverslips were removed from the wells at 3, 24, 48, and 72 h of co-cultivation, washed in PBS, fixed in absolute methanol, and stained with Giemsa (Merck, Darmstadt, Germany) or rapid Panoptic stain (Laborclin, Pinhais, SP, Brazil).^18,29^ The slides were analysed using a standard optical microscope (Primo Star Zeiss), and images were captured using AxioVision 4.8. Image adjustment (brightness, contrast, and scale) and analysis were performed on ImageJ.

To determine the percentages of trophozoites with *L. **amazonensis***, trophozoites containing microbeads, and the average number of parasites or microbeads per trophozoite, 200 trophozoites were counted per coverslip. All counts were made at 1,000× magnification using optical and fluorescence microscopes.^29^

*Viability of *L. **amazonensis*** and *Acanthamoeba* in co-cultures* - For *L. **amazonensis*** viability assessment, parasites were co-cultured for 3 h in RPMI or PYG medium at ratios of 1:10 or 1:20 (trophozoite: promastigotes). Then, the cultures were washed three times with 1× PBS, and trophozoites were lysed with 0.04% sodium dodecyl sulphate in PBS. The cell suspension was passed through seven passages using a 30-G needle in sterile 1-mL syringes.^14^ After the suspension was centrifuged (100 *g*) for 10 min, the supernatant was collected and centrifuged (800 *g*) for 10 min. The pellet containing the *L. **amazonensis*** intracellular forms was resuspended in RPMI medium + 10% FBS and seeded into 24-well plates with RPMI medium + 10% FBS for differentiation into promastigotes at 26ºC. The cultures were quantified daily using a Neubauer chamber over 10 days.^14^,^30^,^31^

For amoeba viability assessment, 3 × 10^5^ trophozoites suspended in either fresh RPMI or PYG were plated in 24-well plates containing 13-mm diameter coverslips. These were incubated in several time periods (3, 24, 48, and 72 h), and either at 26ºC or 34ºC. Adherent amoeba were counted in 20 random fields per coverslip.^14,29^ The percentage of viable amoeba co-cultured with promastigotes is depicted as a percentage of the control (trophozoites cultured without promastigotes).

*Fluorescence and confocal microscopy* - *Acanthamoeba* with *L. **amazonensis***-GFP or microbeads were co-cultured at 26ºC at a 1:10 ratio (trophozoite: promastigotes or microbeads) in 24-well plates with 13-mm diameter coverslips in either PYG medium + 10% FBS, or in RPMI medium + 10% FBS. Parasites were fixed in 4% paraformaldehyde after the coverslips were removed, then washed with 1× PBS and permeabilised with Triton-X (0.3%) for 1 min. The coverslips were then stained with Celltracker (diluted 1:1,000 in 1× PBS) for 30 min to label the cytoplasm (Thermo Fisher Waltham, MA, USA), followed by 4',6-diamino-2-phenylindole (DAPI) (diluted 1:1000 in 1× PBS) for 40 min to label the nucleus (Thermo Fisher), and washed with 1× PBS. Lastly, the coverslips were placed onto 4 µL of VectaShield mounting medium.^32^ The slides were then analysed under a fluorescence microscope and a confocal microscope with Airyscan mode (Zeiss LSM880 Airyscan AG, Germany) was used for DAPI excitation (emission filter 450/40 nm), green (FITC) excitation (emission filter 510/20 nm), and Celltracker Red excitation (emission filter 620/30 nm), at 63× oil immersion objective, with zoom 1x. Images were captured using ZEN (Carl Zeiss Microscopy GmbH) software. Image adjustment (brightness, contrast, and scale) was performed using Fiji 3D Script/Vaa3D.

*Live microscopy* - The analysis of the real-time interaction of *Acanthamoeba* trophozoites with *L. **amazonensis***-GFP promastigotes was carried out in a 16-well chamber slide system (LabTek, São Paulo, SP, Brazil), in RPMI medium + 10% FBS, at ratio of 1:10 (trophozoite: promastigotes) and incubated at 26ºC for 24 h. The recordings were performed under an inverted confocal microscope with Airyscan mode (Zeiss LSM880 Airyscan AG, Germany), at 20× oil immersion objective. Images were captured using ZEN (Carl Zeiss Microscopy GmbH) software. Image adjustment (brightness, contrast, and scale) was performed using Fiji.

*Scanning electron microscopy (SEM)* - Topological changes were examined. To this end, 1 × 10^5^ *A. **castellanii*** trophozoites and 1 × 10^6^ *L. **amazonensis*** promastigotes (ratio 1:10 trophozoite: promastigotes) were plated on 13-mm coverslips and, after 2 h, fixed in 2.5% glutaraldehyde (Electron Microscopy Sciences, Hatfield, PA, USA) in 0.1M PBS. After washing with PBS, the samples were post-fixed in 1% osmium tetroxide (1 h) (Electron Microscopy Sciences, Hatfield, PA), sequentially dehydrated in ethanol, washed, brought to the critical point dryer (Balzers CPD-030), and covered with Au, using Sputter Coater (Balzers SCD-050).^33^ Lastly, the samples were analysed using a SEM (JEOL JSM-5800LV), operating at a standard accelerating voltage of 10 kV. Image adjustment (brightness, contrast, and scale) was performed using Fiji.

*Statistical analysis* - The experiments were repeated at least three times independently. The mean and standard deviation (SD) were calculated for all assays, and the analysis was performed using GraphPad Prism version 8.1. Statistical comparisons between the two ratios (1:10 and 1:20) were performed at each time point (3, 24, and 48 hours) in the respective medium using Student's *t*-test. In addition, the percentage of viable amoeba at ratios (1:10 and 1:20) was compared with the control group (only trophozoites). Differences were considered statistically significant when *p* ≤ 0.05.

## RESULTS

**Fig. 1:**
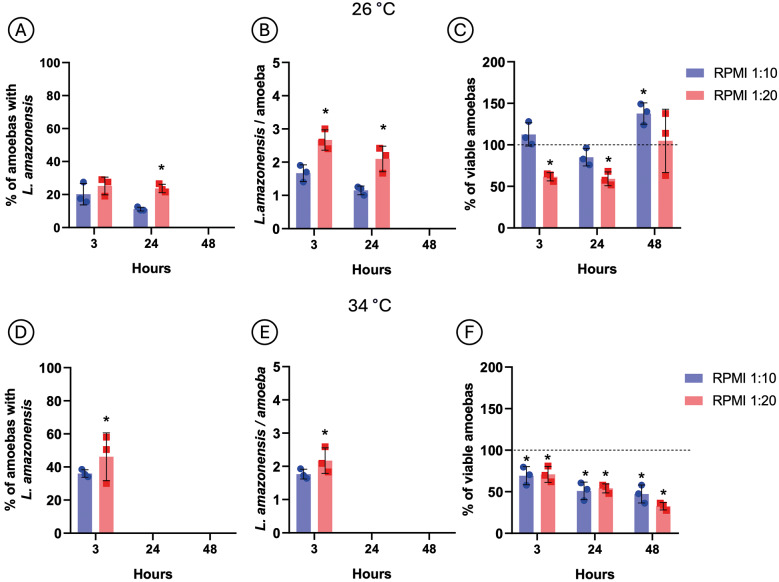
interaction between *Acanthamoeba* trophozoites and *Leishmania **amazonensis*** promastigotes. Trophozoites and promastigotes co-cultured at 26ºC (A, B, and C) and 34ºC (D, E, and F) in RPMI medium, at 3, 24, and 48 h, at ratios of 1:10 and 1:20 (trophozoite: promastigotes). The percentage of viable amoebas is relative to the control group culture without promastigotes (dotted line) (C, F). Data are representative of one of three experiments, performed in triplicate, and values are expressed as mean ± standard deviation (SD). Statistical significance for the two ratios (1:10 and 1:20) investigated at each time point (3, 24, and 48 h) is indicated by *, p ≤ 0.05.

*Uptake assays: interaction between *Acanthamoeba* trophozoites and *L. **amazonensis*** promastigotes* - Firstly, we have confirmed that both microorganisms proliferate effectively in the culture medium commonly used for their cultivation; RPMI medium for cultivating *L. **amazonensis*** promastigotes, and PYG medium for culturing *Acanthamoeba* trophozoites, both at 26ºC [Supplementary data (Fig. 1)]. However, the conditions for *L. **amazonensis*** infection in macrophages *in vitro* require incubation at 34ºC. Consequently, although *Acanthamoeba* does not proliferate at 34ºC [Supplementary data (Fig. 1)], we also chose to evaluate the co-culture of the protozoans at this temperature. The amoeba remains viable for up to 72 h at 34ºC, predominantly in the trophozoite stage, before entering early encystment. We reasoned that this transitional physiological state could still allow for interaction and potential infection by *L. **amazonensis*** promastigotes.

**Fig. 2:**
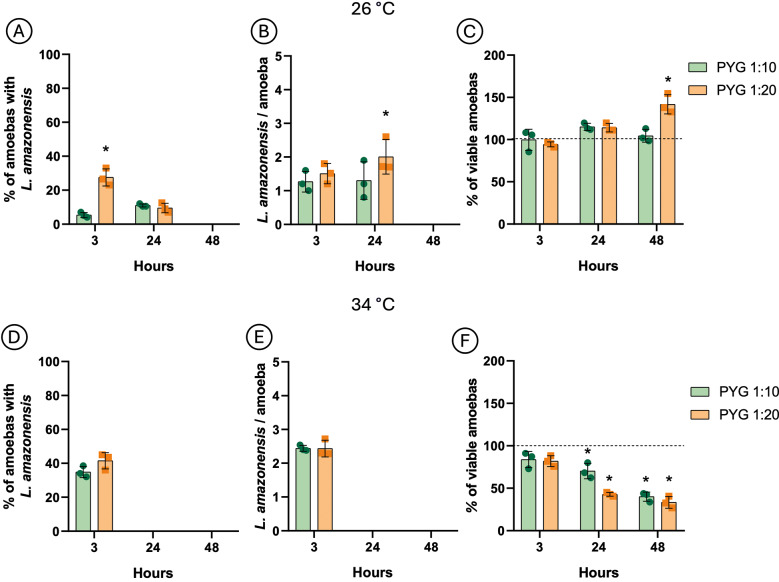
interaction between *Acanthamoeba* trophozoites and *Leishmania **amazonensis*** promastigotes. Trophozoites and promastigotes were co-cultured at 26ºC (A, B, and C) and 34ºC (D, E, and F) in PYG medium + 10% foetal bovine serum (FBS), at 3, 24, and 48 h and ratios of 1:10 and 1:20 (trophozoite: promastigotes). The percentage of viable amoebas is relative to the control group culture without promastigotes (dotted line) (C, F). Data are representative of one of three experiments, performed in triplicate, and values are expressed as mean ± standard deviation (SD). Statistical significance for the two ratios (1:10 and 1:20) investigated at each time point (3, 24, and 48 h) is indicated by *, p ≤ 0.05.

The co-culture between trophozoites and promastigotes was performed at ratios of 1:10 and 1:20 (trophozoite: promastigotes), using either RPMI (Fig. 1) or PYG (Fig. 2). Regarding incubations in RPMI, *L. **amazonensis*** were only viable up to 24 h at 26ºC, with trophozoites (Fig. 1A). Interestingly, at 34ºC, it only survived up to a 3 h incubation period (Fig. 1D). The trophozoite: promastigote ratio did not affect the amoeba viability or the total number of amoeba with internalised promastigotes (Fig. 1A, C). However, this ratio affected the number of promastigotes internalised per trophozoite at both temperatures, with a slight increase observed at a 1:20 (trophozoite: promastigotes) ratio (Fig. 1B).

The co-culture was also performed in PYG medium (Fig. 2), in which we found that *L. **amazonensis*** could survive only up to 24 h within amoeba at 26ºC (Fig. 2A, D). Amoeba viability was only affected at 34ºC (Fig. 2C), and the trophozoite: promastigotes ratio impacted only the total amoeba with internalised *L. **amazonensis*** at a 3 h incubation period at 26ºC (Fig. 2A).

**Fig. 3:**
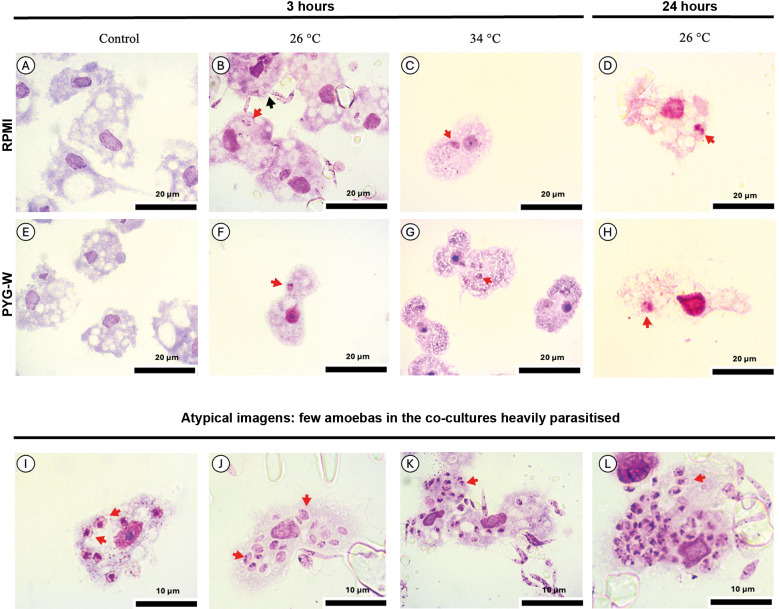
*Acanthamoeba* trophozoites and *Leishmania **amazonensis*** were co-cultured in RPMI or PYG medium. Trophozoites cultured without promastigotes (A, E); trophozoites co-cultured with promastigotes at 26ºC (B, F) and at 34ºC (C, G) for 3 h; trophozoites co-cultured with promastigotes at 26ºC for 24 h (D, H); atypical images: a few amoebas in the co-cultures were heavily parasitised, as indicated by the presence of a large number of intracellular *L. **amazonensis*** (I, J, K, L). Cells were stained with Giemsa. Red arrows: trophozoites containing intracellular *L. **amazonensis*** and black arrows: promastigote interacting with trophozoites through the flagellum.

This interaction is represented in the photomicrographs of the kinetics of co-cultures in Fig. 3, which indicates the intracellular forms of *L. **amazonensis*** over time (Fig. 2A-H). Most of the internalised forms of *L. **amazonensis*** became rounded and shortened after 3 h of co-culture, and apparently lost their flagellum (Fig. 3), as it occurs with intracellular amastigotes. In addition, a very limited number of trophozoites were **heavily *parasitised*** with *L. **amazonensis*** (Fig. 3I-L).

Altogether, based on the rates of *L. **amazonensis*** internalisation by the amoeba and amoeba viability, the most optimal condition for the co-culture of promastigotes and trophozoites is in RPMI medium at 26ºC at the ratio of 1:10.

**Fig. 4:**
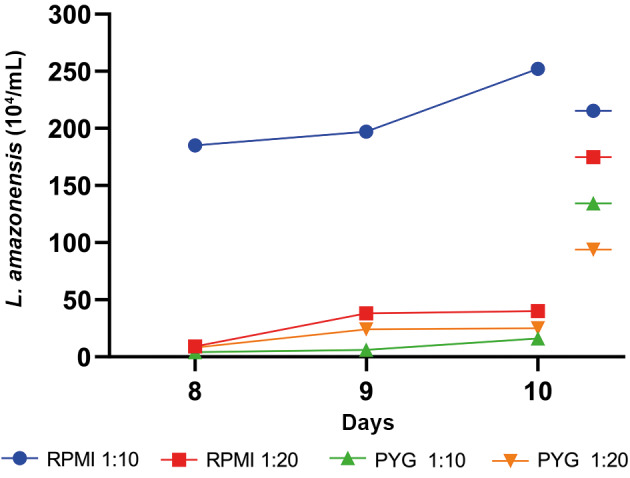
detection of *Leishmania **amazonensis*** viability by proliferation curves of promastigotes recovered from co-cultures. Intracellular *L. **amazonensis*** forms were recovered from trophozoites co-cultured in RPMI or PYG medium, at ratios of 1:10 and 1:20 (trophozoite: promastigotes), after 3 h. The recovered intracellular forms were seeded in 24-well plates in RPMI medium for differentiation into promastigotes at 26ºC and counted using a Neubauer chamber over 10 days.

*Viability of internalised forms of *L. **amazonensis**** - Intracellular forms of *L. **amazonensis*** were successfully isolated from trophozoites after 3 h co-cultivation in RPMI medium, and differentiated into proliferating promastigotes, confirming *L. **amazonensis*** viability (Fig. 4). However, after 24 h of co-cultivation, no *L. **amazonensis*** were recovered from within the trophozoites. The trophozoites co-cultured in PYG medium also harboured viable *L. **amazonensis***, but their ability to differentiate into promastigotes was reduced (Fig. 4).

*Optical, fluorescence, confocal microscopy, and live microscopy* - Initially, to evaluate our staining methods for *Acanthamoeba* cultures, commercially available FITC microbeads were used as a reference^34^ [Supplementary data (Figs 2-3)]. The uptake, incorporation, and retention of microbeads were visualised in fluorescence microscopy. As expected, trophozoites were able to uptake microbeads, and after 3 h, up to 50% of amoebas contained microbeads (2-5 microbeads per amoeba) at both temperatures (26ºC and 34ºC) and culture medium (RPMI and PYG) [Supplementary data (Fig. 2)].

**Fig. 5:**
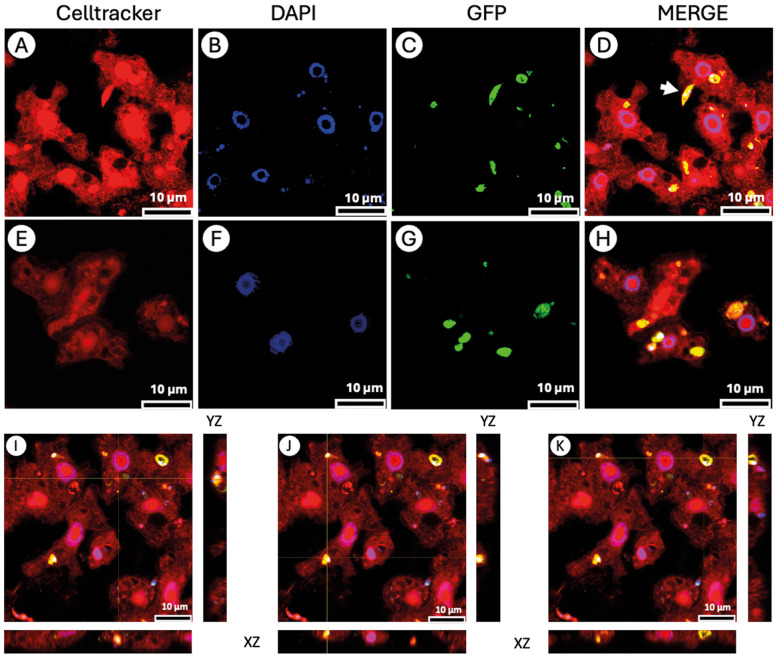
confocal microscopy of co-cultures of *Acanthamoeba* trophozoites and *Leishmania **amazonensis***-green fluorescent protein (GFP) promastigotes. Confocal microscopy of co-cultures of *Acanthamoeba* trophozoites and *L. **amazonensis***-GFP promastigotes stained with Celltracker (cytoplasm) (A and E), and DAPI (nucleus) (B and F), *L. **amazonensis***-GFP (C and G), and Merge (D and H). Orthogonal cutting confocal microscopy of *Acanthamoeba* trophozoites and *L. **amazonensis***-GFP promastigotes co-cultures, stained with Celltracker and DAPI (L, I, and J). White arrow: promastigote interacting with trophozoite through the flagellum.

Subsequent assays using *L. **amazonensis***-GFP revealed that approximately 27% of amoebas were found to contain the parasite, with an *L. **amazonensis***/trophozoite rate of 0.8-2.8/amoeba after 3 h, which was similar to what was obtained with the *L. **amazonensis*** wild-type strain (Fig. 1). Using fluorescence microscopy, it was possible to observe acanthopodia in the trophozoites as well as the interaction of *L. **amazonensis***-GFP promastigotes with trophozoites through their flagellum (Fig. 5). Through orthogonal visualisation of the XYZ axes using a confocal microscope (Fig. 5I-J, K), it was confirmed that *L. **amazonensis***-GFP was located inside the amoeba instead of overlapping with it. This was corroborated by the 3D processing of 30 interaction planes [Supplementary data (Figs 4-5, Movie 1)].

In addition, time-lapse microscopy demonstrated that the number of *L. **amazonensis***-GFP decreased after 6 and 24 h of interaction compared with 3 h. In addition, this confirmed that the amoeba phagocytosed *L. **amazonensis***-GFP promastigotes via its acanthopodia [Supplementary data (Movies 2-3)].

**Fig. 6:**
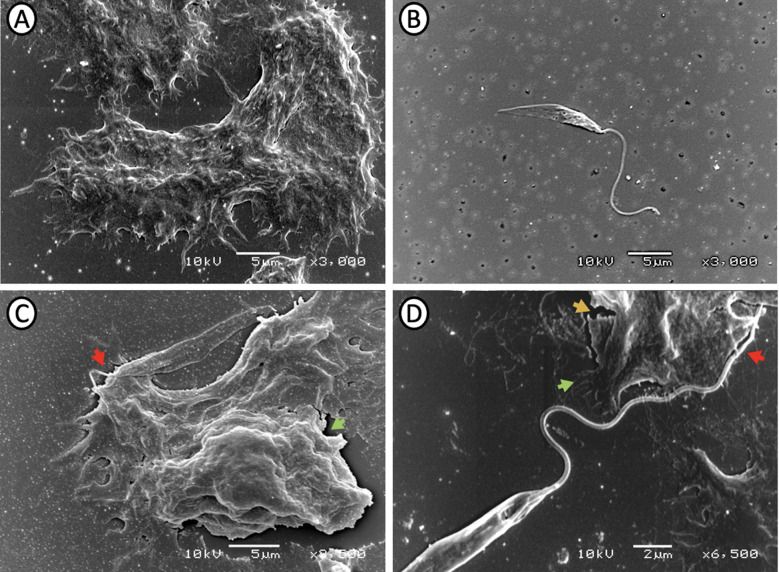
scanning electron microscopy (SEM) of *Acanthamoeba* trophozoites and *Leishmania **amazonensis*** promastigotes. Trophozoite cultured without promastigotes (control) (A), promastigote cultured without trophozoites (control); promastigote (B); interaction between trophozoites and promastigotes (C and D). green arrows: trophozoites, red arrows: promastigotes attached to trophozoite, and yellow arrow: trophozoite surface damage.

*Topological analysis and parasite morphological changes in co-cultures* - To examine the surface interactions between *L. **amazonensis*** and *Acanthamoeba*, we performed SEM on trophozoites (control) and promastigotes (control), and compared them with parasites co-cultured for 2 h (Fig. 6A-D). We confirmed that promastigotes interacted with trophozoites, primarily via their flagella. Interestingly, the surface of the trophozoites appeared damaged after the interaction with promastigotes, compared with the control (Fig. 6D).

## DISCUSSION

Our results demonstrated the interaction between promastigotes and trophozoites and the presence of *L. **amazonensis*** within the amoeba visually. The amoeba phagocytosed the promastigotes via acanthopodia, which are spine-like structures on the surface used to capture particles.^35^ These results corroborate previous findings in which the interaction between *L. **braziliensis*** amastigotes isolated from a skin lesion and *Acanthamoeba* trophozoites was evaluated by transmission electron microscopy, demonstrating that the amoeba extended acanthopodia within 3 h of co-cultivation.^24^ Similar conclusions were obtained in a recent study using optical microscopy.^25^

Although our results confirm what was reported in the only two articles in the literature so far, our study provides a more detailed morphological and kinetic characterisation of the interaction between these protozoans, combining optical, fluorescence, electron, confocal, and live-video microscopy. Our three-dimensional approach overcomes the limitation of two-dimensional microscopy by conclusively confirming the intracellular localisation of *Leishmania* within amoeba. As a novel contribution, our group has captured protozoan interactions using SEM and observed membrane damage on the trophozoite during interaction with *L. **amazonensis***. Furthermore, combining parasite counting with optical microscopy and real-time microscopy provides a better overview of what happens during the interaction. Interestingly, our results confirm that *Acanthamoeba* trophozoites can eliminate *L. **amazonensis*** rather quickly.

It should be noted that the population of *L. **amazonensis*** promastigotes used in our work exhibited persistent infection in RAW 264.7-derived macrophages after 72 h (data not shown). This indicates that these parasites were able to persist within macrophages, unlike what occurred in co-cultures with trophozoites.

Interestingly, most *L. **amazonensis*** promastigotes interact with trophozoites via their flagellum, a process that also occurs when promastigotes infect cells of the mammalian mononuclear phagocyte system.^3,36^ The presence of conserved immune receptors explains this interaction. For instance, amoeba express TLR-5, a pattern recognition receptor that binds flagellin, analogous to its function in mammalian phagocytic cells.^37^,^38^ Taken together, the results indicate that *Leishmania* can penetrate the amoeba both actively and passively via phagocytosis, in a manner comparable to infection of mammalian macrophages.^36^,^39^ The phagocytosed forms of *L. **amazonensis*** become rounded and shortened or lose the flagellum, and resemble amastigotes, the intracellular parasite form observed within the parasitophorous vacuoles of mammalian macrophages. Although it appears that these amastigote-like forms are located within amoeba vacuoles, as previously suggested,^25^ it will only be possible to confirm the localisation of *Leishmania* spp. with future analysis of vacuole markers inside an amoeba.

Regarding the presence of *L. **amazonensis*** within the amoeba, the data indicated that, during the first 3 h of co-culture, intracellular amastigote-like forms remained viable. At this time point, 20% of amoeba contained *L. **amazonensis***, with an infection rate of 1.6 parasites per amoeba. The parasites were successfully isolated from trophozoites and differentiated back into axenic promastigotes, demonstrating their viability inside amoeba. However, no proliferation of *L. **amazonensis*** inside amoeba was detected over longer periods. Indeed, over time, the number of amastigote-like forms decreased, and no parasites were found inside the trophozoites after 48 h. Indeed, this result corroborates previous findings in which researchers, incidentally, observed a similar pattern in a control experiment with *L. tropica*. In their study, internalised *Leishmania* decreased and were completely cleared from the amoeba after 48 h of co-culture.^18^

It is worthwhile to compare the results with those described for mammalian macrophages. The kinetic profile, *i.e.*, a decrease in *L. **amazonensis*** infection rate over time in co-cultures of trophozoites and promastigotes, was not observed in most commonly used cell systems, such as murine peritoneal macrophages, bone marrow cultures, and RAW 264.7-derived macrophages, in which the number of amastigotes increased over time.^6,25^ In any case, the infection process and the fate of intracellular parasites may differ *in vivo*.

The mechanism by which *Acanthamoeba* inactivates and kills *Leishmania* spp. remains unexplored at present. It is recognised that *Acanthamoeba* acts as a phagotrophic predator, processing a well-developed phagosome-associated feeding pathway, and producing oxidative stress through ROS and NO.^16,17^ Additional antimicrobial strategies have been described, including the production of pore-forming toxins, soft metal poisoning, and the exploitation of bacterial motility.^40^,^41^,^42^,^43^ Preliminary data from our laboratory (data not shown), measuring nitrite by the Griess method,^44^ indicated no elevated levels of nitrite in the co-cultures of *Acanthamoeba* trophozoites and *L. **amazonensis*** promastigotes.

The main objective of this study was to characterise in depth the fundamental interaction between *Acanthamoeba* and *L. **amazonensis***. As a novel contribution to the literature, we determined that the optimal experimental condition was RPMI medium at 26ºC. Live microscopy revealed that *L. **amazonensis*** is briefly internalised by *Acanthamoeba*; however, its survival rate decreased after 24 h. Thus, considering that the amoeba can clear *Leishmania* spp. Regarding infection, we suggest that this amoeba model is not relevant for *in vitro* studies, such as drug screening. However, this interaction could serve as a model for studying cellular leishmanicidal mechanisms, *i.e.*, strategies for killing microorganisms. Studies involving *Acanthamoeba* cytolytic mechanisms during the period of interaction with *Leishmania* spp. could reveal valuable information about molecules with potent microbicidal functions.

We do not know whether there is any ecological significance to the *Acanthamoeba*-*Leishmania* spp. interaction, nor whether this occurs in its natural environment. However, *Acanthamoeba* can act both as a predator and as an environmental host for several pathogens. There is a diversity of medically relevant microorganisms that the amoeba can interact with and influence their virulence in the environment. While amoebae can eliminate some microorganisms, they also internalize pathogens like *Legionella* and *Cryptococcus* into their vacuoles, thereby protecting these pathogens and enhancing their virulence.^21^,^35^ When challenged, the amoeba transforms into a remarkably resilient, double-walled cyst. This resistance ensures the environmental persistence of both the protozoan and any intracellular pathogens, allowing *Acanthamoeba* to thrive in virtually any environment.^12^,^13^,^16^

Thus, considering that the *Acanthamoeba* has been isolated from wild mosquitoes *Aedes aegypti*, it is possible that the amoeba interacts with the larval stage of the sandfly vector and, later, within the adult insect stage, interacts with *Leishmania* spp. promastigotes. The hypothesis is that larval stages of sandflies ingest the amoeba found in aquatic environments and biofilms and acquire them in the digestive tract. When adult sandflies take a blood meal from mammals infected by *Leishmania* spp., they ingest infected cells, and the amastigotes transform into procyclic promastigotes that interact with amoebas within the digestive tract. Likewise, adult sandflies can acquire amoeba, creating conditions for rapid interactions once they become infected with *Leishmania* promastigotes. Although the viability of trophozoites in this microenvironment may be limited, a rapid interaction with *Leishmania* spp. could exert some influence on the parasites. Future studies are required to assess *Acanthamoeba*'s role as a natural host for various pathogens.

In conclusion, this study confirms that *Acanthamoeba* can interact and internalise *L. **amazonensis***. Once internalised, the parasites shift into an amastigote-like form and can be cleared by the amoeba host. The application of reverse-engineering approaches, such as omics-based analyses, may help to elucidate the molecular pathways within the amoeba that lead to its leishmanicidal activity. Understanding these mechanisms could open new avenues for exploring host-pathogen interactions and for identifying innovative strategies with potential therapeutic relevance.

## SUPPLEMENTARY MATERIALS

Supplementary data

## Data Availability

The contents underlying the research text are included in the manuscript.

## References

[1] WHO - World Health Organization (2025). Leishmaniasis.

[2] Mann S, Frasca K, Scherrer S, Henao-Martínez AF, Newman S, Ramanan P (2021). A review of leishmaniasis: current knowledge and future directions. Curr Trop Med Rep.

[3] Serafim TD, Coutinho-Abreu IV, Dey R, Kissinger R, Valenzuela JG, Oliveira F (2021). Leishmaniasis: the act of transmission. Trends Parasitol.

[4] Reithinger R, Dujardin JC, Louzir H, Pirmez C, Alexander B, Brooker S (2007). Cutaneous leishmaniasis. Lancet Infect Dis.

[5] Ankeny RA, Leonelli S (2011). What's so special about model organisms?. Stud Hist Philos Sci A.

[6] Bogdan C (2020). Macrophages as host, effector and immunoregulatory cells in leishmaniasis. Cytokine X.

[7] Terreros MJS, de Luna LAV, Giorgio S (2019). Evaluation of antileishmanial drugs activities in an ex vivo model. Parasitol Int.

[8] O'Keeffe A, Hale C, Cotton JA, Yardley V, Gupta K, Ananthanarayanan A (2020). Novel 2D and 3D assays for anti-leishmanial drugs. Microorganisms.

[9] Gupta AK, Das S, Kamran M, Ejazi SA, Ali N (2022). Pathogenicity and virulence of *Leishmania* - interplay of virulence factors with host defenses. Virulence.

[10] Montagnes D, Roberts E, Lukeš J, Lowe C (2012). The rise of model protozoa. Trends Microbiol.

[11] Geres LF, Sartori E, Neves JMS, Miguel DC, Giorgio S (2024). Amebicides against *Acanthamoeba castellanii*: the impact of organism models used in amebicide assays. Parasitologia.

[12] Zhang H, Cheng X (2021). Various brain-eating amoebae: the protozoa, the pathogenesis, and the disease. Front Med.

[13] Lacerda AG, Lira M (2021). *Acanthamoeba keratitis*: a review of biology, pathophysiology and epidemiology. Ophthalmic Physiol Opt.

[14] de Faria LV, do Carmo PHF, da Costa MC, Peres NTA, Chagas IAR, Furst C (2020). Acanthamoeba castellanii as an alternative interaction model for the dermatophyte Trichophyton rubrum. Mycoses.

[15] Fukaya S, Masuda L, Takemura M (2023). Analysis of morphological changes in the nucleus and vacuoles of *Acanthamoeba castellanii* following Giant virus infection. Microbiol Spectr.

[16] Mungroo MR, Siddiqui R, Khan NA (2021). War of the microbial world: *Acanthamoeba* spp. interactions with microorganisms. Folia Microbiol.

[17] Rayamajhee B, Subedi D, Peguda HK, Willcox MD, Henriquez FL, Carnt N (2021). A systematic review of intracellular microorganisms within *Acanthamoeba* to understand potential impact for infection. Pathogens.

[18] Winiecka-Krusnell J, Dellacasa-Lindberg I, Dubey JP, Barragan A (2009). *Toxoplasma gondii*: uptake and survival of oocysts in free-living amoebae. Exp Parasitol.

[19] Boratto PVM, Oliveira GP, Machado TB, Andrade ACSP, Baudoin JP, Klose T (2020). Yaravirus: a novel 80-nm virus infecting *Acanthamoeba castellanii*. Proc Natl Acad Sci USA.

[20] Boratto PVM, Oliveira GP, Abrahão JS (2022). "Yaraviridae": a proposed new family of viruses infecting *Acanthamoeba castellanii*. Arch Virol.

[21] Carvalho JHS, Nascimento JKC, Silva KGV, Silveira Neto S, Macedo AT, França HL (2023). Yeast-amoeba interaction influences murine cryptococcosis. Microbes Infect.

[22] Ferreira MS, Mendoza SR, Gonçalves DS, Rodríguez-de la Noval C, Honorato L, Nimrichter L (2022). Recognition of cell wall mannosylated components as a conserved feature for fungal entrance, adaptation and survival within trophozoites of *Acanthamoeba castellanii* and murine macrophages. Front Cell Infect Microbiol.

[23] Ahmed K (2014). Could *Acanthamoeba* have hosted and trained *Leishmania* to evade innate immune response?. Med Hypotheses.

[24] Campo-Aasen I, Convit J, Perez-Suarez E, Gallinotto ME (1988). In vitro interaction of Acanthamoeba castellanii with Leishmania braziliensis. Cell Mol Biol.

[25] Santos HLC, Pereira GL, Reis RB, Rodrigues IC, d'Avila CM, Vidal VE (2024). Using *Acanthamoeba* spp. as a cell model to evaluate *Leishmania* infections. PLoS Negl Trop Dis.

[26] Chang KP, Reed SG, McGwire BS, Soong L (2003). *Leishmania* model for microbial virulence: the relevance of parasite multiplication and Patho antigenicity. Acta Trop.

[27] Costa SS, Golim MA, Rossi-Bergmann B, Costa FTM, Giorgio S (2011). Use of *in vivo* and *in vitro* systems to select *Leishmania amazonensis* expressing green fluorescent protein. Korean J Parasitol.

[28] Corsaro D, Mrva M, Colson P, Walochnik J (2024). Validation and redescription of *Acanthamoeba* terricola Pussard, 1964 (Amoebozoa: Acanthamoebidae). Eur J Protistol.

[29] Barbosa AM, Costa SS, da Rocha JR, Montanari CA, Giorgio S (2015). Evaluation of the leishmanicidal and cytotoxic effects of inhibitors for microorganism metabolic pathway enzymes. Biomed Pharmacother.

[30] Mendes B, Minori K, Consonni SR, Andrews NW, Miguel DC (2022). Causative agents of American Tegumentary Leishmaniasis are able to infect 3T3-L1 adipocytes *in vitro*. Front Cell Infect Microbiol.

[31] Rayamajhee B, Willcox M, Henriquez FL, Vijay AK, Petsoglou C, Shrestha GS (2024). The role of naturally acquired intracellular *Pseudomonas aeruginosa* in the development of *Acanthamoeba* keratitis in an animal model. PLoS Negl Trop Dis.

[32] Garajová M, Mrva M, Vaškovicová N, Martinka M, Melicherová J, Valigurová A (2019). Cellulose fibrils formation and organisation of cytoskeleton during encystment are essential for *Acanthamoeba* cyst wall architecture. Sci Rep.

[33] Albuquerque P, Nicola AM, Magnabosco DAG, Derengowski LS, Crisóstomo LS, Xavier LCG (2019). A hidden battle in the dirt: soil amoebae interactions with *Paracoccidioides* spp.. PLoS Negl Trop Dis.

[34] Elloway EAG, Bird RA, Hewitt CJ, Kelly SL, Smith SN (2006). Characterization of Acanthamoeba-microsphere association by multiparameter flow cytometry and confocal microscopy. Cytometry A.

[35] Siddiqui R, Khan NA (2012). Biology and pathogenesis of *Acanthamoeba*. Parasit Vectors.

[36] Vannier-Santos MA, Martiny A, Souza W (2002). Cell Biology of *Leishmania* spp.: invading and evading. Curr Pharm Des.

[37] Gonçalves DS, Ferreira MS, Gomes KX, Rodríguez-de La Noval C, Liedke SC, da Costa GCV (2019). Unravelling the interactions of the environmental host *Acanthamoeba castellanii* with fungi through the recognition by mannose-binding proteins. Cell Microbiol.

[38] Nasher F, Wren BW (2023). Flagellin O-linked glycans are required for the interactions between *Campylobacter jejuni* and *Acanthamoeba castellanii*. Microbiology.

[39] Martínez-López M, Soto M, Iborra S, Sancho D (2018). *Leishmania* hijacks myeloid cells for immune escape. Front Microbiol.

[40] Michalek M, Sönnichsen FD, Wechselberger R, Dingley AJ, Hung CW, Kopp A (2013). Structure and function of a unique pore-forming protein from a pathogenic *Acanthamoeba*. Nat Chem Biol.

[41] Yabrag A, Ullah N, Baryalai P, Ahmad I, Zlatkov N, Toh E (2025). A new understanding of *Acanthamoeba castellanii*: dispelling the role of bacterial pore-forming toxins in cyst formation and amoebicidal actions. Cell Death Discov.

[42] German N, Doyscher D, Rensing C (2013). Bacterial killing in macrophages and amoeba: do they all use a brass dagger?. Future Microbiol.

[43] de Schaetzen F, Fan M, Alcolombri U, Peaudecerf FJ, Drissner D, Loessner MJ (2022). Random encounters and amoeba locomotion drive the predation of *Listeria monocytogenes* by *Acanthamoeba castellanii*. Proc Natl Acad Sci USA.

[44] Karaś MA, Turska-Szewczuk A, Marczak M, Jaszek M, Janczarek M, Dworaczek K (2018). A mutation in the *Mesorhizobium loti oatB* gene alters the physicochemical properties of the bacterial cell wall and reduces survival inside *Acanthamoeba castellanii*. Int J Mol Sci.

