# Supplementary material for: Uptake and survival of Leishmania amazonensis in Acanthamoeba: an adequate model organism?
Source: Mem Inst Oswaldo Cruz. 2026 Jun 19;121:e250253. doi: 10.1590/0074-02760250253 (PMC13282119; doi:10.1590/0074-02760250253)
Supplement: Supplementary data [file 1678-8060-mioc-121-e250253-s1.pdf]

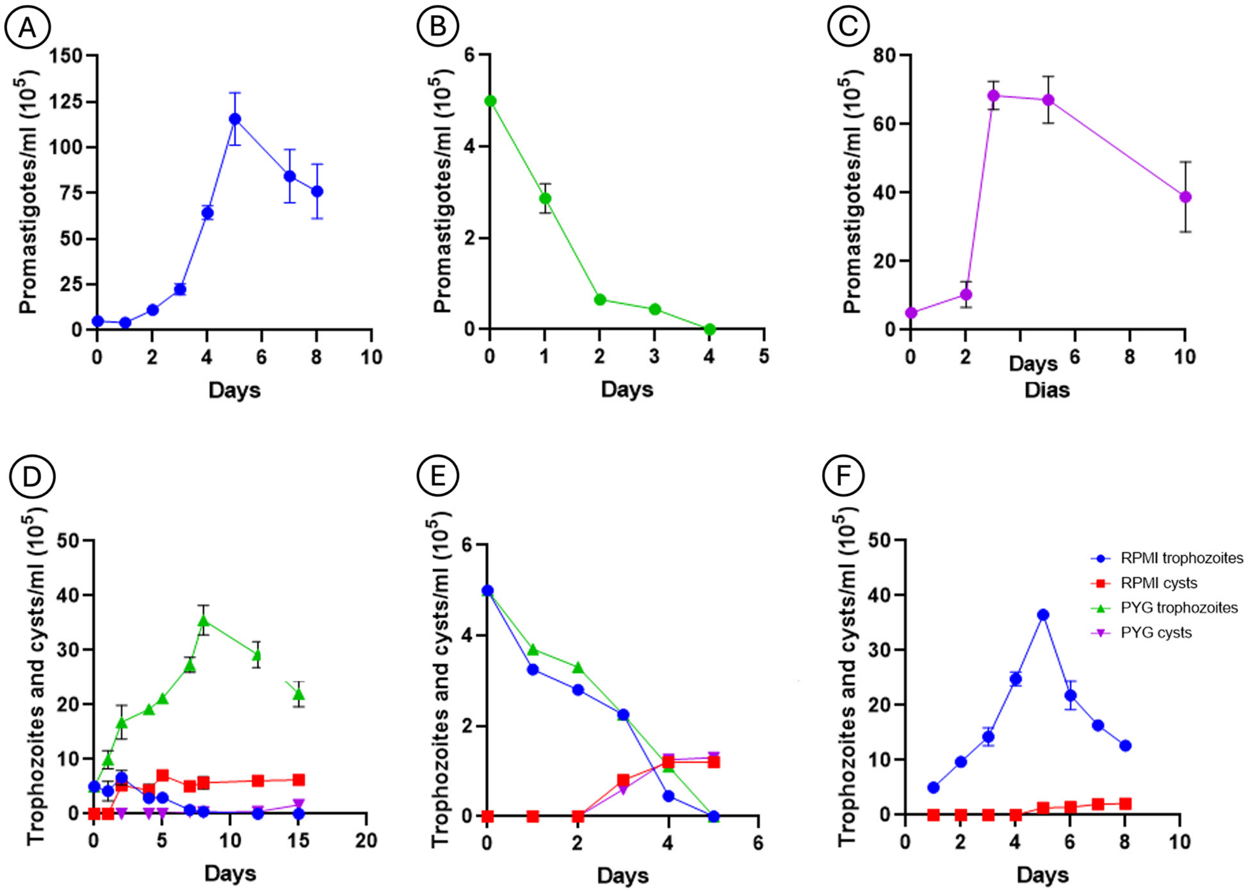

Fig. 1: proliferation curves of *Leishmania amazonensis* promastigotes and *Acanthamoeba*. Approximately 5 x 10^5 promastigotes/mL or trophozoites/mL were cultured separately in different medium and temperatures and counted in a Neubauer chamber. Number of promastigotes cultured in: RPMI medium at 26ºC (A); PYG medium at 26ºC (B); PYG medium BS at 26ºC (C). Number of trophozoites and cysts in different medium at 26ºC (D), 37ºC (E), and 34ºC (F).

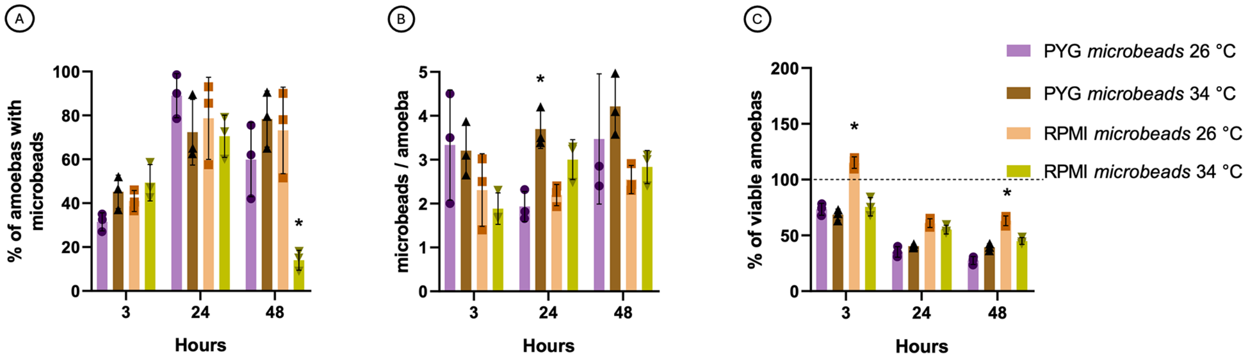

Fig. 2: interaction between *Acanthamoeba* trophozoites and microbeads. Trophozoites and fluorescent microbeads were maintained at 26ºC or 34ºC (A, B, C) in RPMI or PYG medium, at 3, 24, and 48 h, at a ratio of 1:10. Percentage of viable amoebas is relative to the control group culture without promastigotes (dotted line) (C). Data are representative of one of three experiments, performed in triplicate, and values are expressed as mean ± standard deviation (SD). Statistical significance for the two ratios (1:10 and 1:20) investigated at each time point (3, 24, and 48 h) is indicated by *, $p \leq 0.05$.

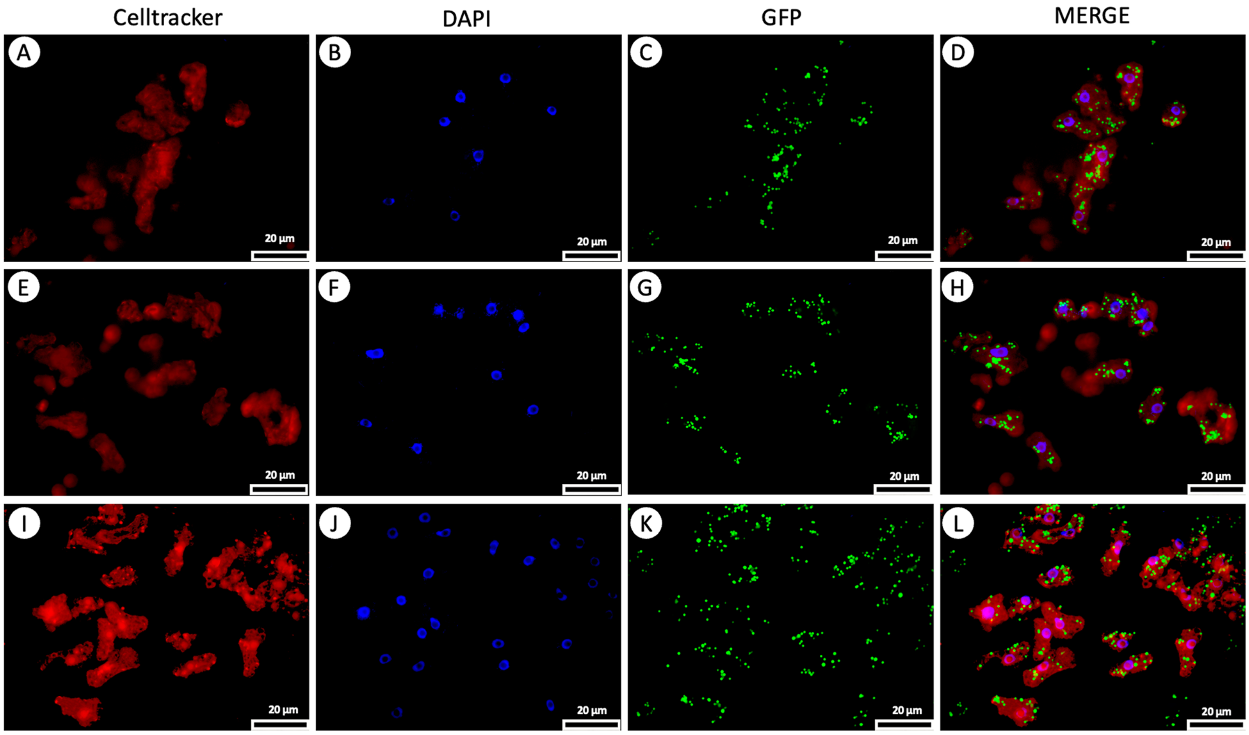

Fig. 3: fluorescence microscopy of trophozoites and microbeads. Trophozoites interacting with fluorescent microbeads were stained with Celltracker (cytoplasm) and DAPI (nucleus), Celltracker (A, E, and I), DAPI (B, F, and J), fluorescent green microbeads (C, G, and K), and Merge (D, H, and L).

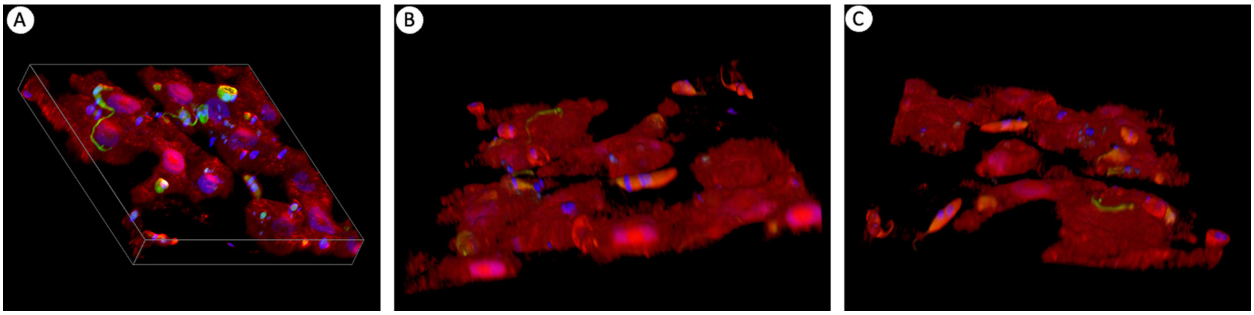

Fig. 4: three-dimensional volumetric (3D model) reconstruction of the sample of co-cultures obtained through confocal microscopy. Trophozoites interacting with *Leishmania amazonensis*-GFP were stained with Celltracker and DAPI.

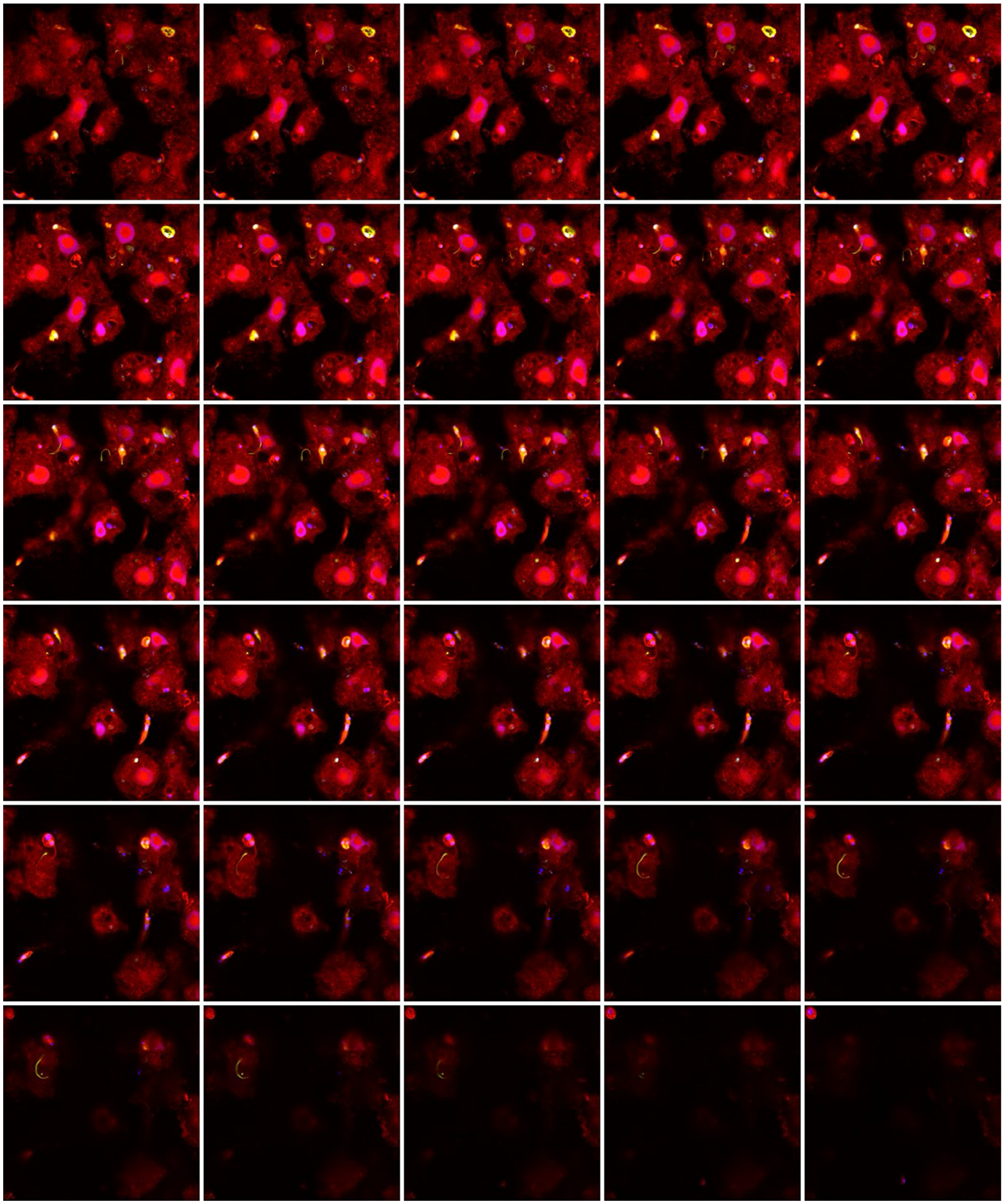

Fig. 5: plate of 30 views of *Acanthamoeba* trophozoites and *Leishmania amazonensis* promastigotes co-culture on confocal microscopy.

MOVIE 1
click here to access

3D visualisation of the interaction between *Acanthamoeba* trophozoites and *Leishmania amazonensis* promastigotes.

MOVIE 2
click here to access

Time-lapse video showcasing the dynamic interaction between *Acanthamoeba* trophozoites and *Leishmania amazonensis* promastigotes over a 24 h period. Observe the movement, behaviour, and possible phagocytosis events in this captivating micro-scale interaction.

MOVIE 3
click here to access

Time-lapse video showcasing a brief dynamic interaction between *Acanthamoeba* trophozoites and *Leishmania amazonensis* promastigotes.
