# Supplementary material for: Uptake and survival of Leishmania amazonensis in Acanthamoeba: an adequate model organism?
Source: Mem Inst Oswaldo Cruz. 2026 Jun 19;121:e250253. doi: 10.1590/0074-02760250253 (PMC13282119; doi:10.1590/0074-02760250253)
Supplement: Peer review history

# Reviewer report: FIRST REVIEW ROUND - REVIEWERS' COMMENTS

## REVIEWER #1

This manuscript investigates the use of *Acanthamoeba* as a model to study *Leishmania **amazonensis*** infection. The premise is interesting given the cellular and functional similarities between these free-living phagocytic amoebae and mammalian macrophages, the primary host cells for *Leishmania*. In specific contexts, it could serve as an alternative to complex or ethically demanding animal procedures. The topic has limited precedent, with only two major previous experimental studies directly focusing on the interaction of *Acanthamoeba* and *Leishmania*: Campos-Aisen in 1988 (PMID: 3208254), and a recent paper by Santos et al., 2024 (doi:10.1371/journal.pntd.0012517), who analyzed *Acanthamoeba* infectivity by four *Leishmania* species (including *L. **amazonensis***) using light and fluorescence microscopy.

Considering this established context, I have three overall considerations of the manuscript: 1) It is crucial to define its novel contribution to avoid the perception of merely confirming or extending prior findings. For instance, the quantitative analysis of infection rates, combined with confocal and live microscopy, offers a deeper, more detailed examination of the interaction than previously reported. 2) The stated objective of evaluating *A. **castellanii*** as a model organism seems somewhat broad, considering the work did not apply the model to a mechanism investigation. In my view, the presented assays are descriptive, aimed at first understanding the biology of the interaction itself. 3) The manuscript needs a clearer logical story. The reasons for key experimental choices (like the temperatures used or why PCR was needed) are not well explained. The manuscript also requires revisions to English grammar, syntax, and style to improve sentence flow and ensure precise scientific expression.

Specific points to be addressed to strengthen the manuscript are detailed below:

Abstract:

1. Lines 28-31: Abstract opening sentence is factually correct, but it is a general statement that is dispensable. I recommend that the authors begin by directly stating the research challenge (e.g., "Research on *Leishmania* pathogenesis, drug, and vaccine discovery remains dependent mainly on mammalian models, which are complex and ethically challenging"), then introduce their proposed solution using a simpler, accessible system.

2. Line 33: It is known that the interaction between *Acanthamoeba* and *L. **amazonensis*** is not fully understood; thus, the study provided a more detailed morphological and kinetic characterization of the infection of this protozoan. To establish a more logical link between the knowledge gap and the methodology employed, I suggest that the authors refine their objective to better align with their experimental approach.

3. Line 45: The term "not relevant" carries an undervaluing tone. I suggest reframe the conclusion avoiding this term, focusing instead on the model's specific suitability. The idea can be preserved by stating that it is less suited for sustained infection studies, such as drug screening, while highlighting its value for investigating leishmanicidal mechanisms.

4. Lines 31,33,35,44: Revise the binomial designation *A. castelllanii*, considering the comment #5 below.

Introduction

5. The manuscript refers to the model organism as *Acanthamoeba **castellanii***. However, not all cited studies or general statements about the genus are specific to this species. To improve taxonomic accuracy and readability, I recommend using *Acanthamoeba* for general statements (e.g., lines 89, 93, 97, 103, 108), reserving the full binomial nomenclature *Acanthamoeba **castellanii*** (or *A. **castellanii*** after first use) specifically when citing work that indeed used this species. This must also be applied to the article title.

6. Line 101: The mention of Yaravirus should be supported by citations of its foundational descriptions. I recommend including the seminal work by Boratto et al. 2020 (doi: 10.1073/pnas.2001637117) or a subsequent detailed analysis such as Boratto et al. 2022 (doi: 10.1007/s00705-021-05326-1)

7. Lines 118-124: The Objectives section requires reformulation. It currently mixes a broad objective with methodological details (e.g., the justification for using *L. **amazonensis***) that are better placed in the Methods section. Please revise this section to focus on the study's core aim (see Comment #2).

Material and Methods

8. Line 135: The strain ATCC 30010 has been historically classified as *Acanthamoeba **castellanii***. A recent taxonomic revision, however, has reclassified it as *A. terricola* (Corsaro et al., 2024; doi:10.1016/j.ejop.2024.126091). To ensure taxonomic accuracy while maintaining clarity with the historical literature, I recommend that the authors note the reclassification in this section. Throughout the rest of the manuscript, they could primarily use the genus name (*Acanthamoeba*) or the strain designation (ATCC 30010) to avoid confusion.

9. The standard medium used for the axenic culture of *Acanthamoeba* is designated as PYG (Peptone-Yeast Extract-Glucose). Please remove the 'W' throughout the manuscript to align with the conventional nomenclature in the field.

10. Line 179-182: The Mat & Met section 2.4 requires clarification. The text abruptly shifts from describing the protocol for lysing amoebae to recover *Leishmania* (lines 169-178) to a sentence about counting 'adherent and intact amoebas' (lines 179-180) without specifying that this was a separate, parallel experiment. I suggest inserting a transitional phrase such as 'In a separate set of wells,' or 'For amoeba viability assessment,' at the beginning of line 179. Furthermore, the reference [25] (line 180) does not refer to the *Acanthamoeba* analysis of viability.

Results

11. Line 250-255 and Fig S1: The opening statement of the results presents a known fact without providing novel insight. Furthermore, the citations (#30, #31) are dispensable here, as this section should focus on presenting new findings rather than referencing established methods. The primary concern, however, is the rationale for the temperature choices in the interaction assays. The data in Figure S1F clearly show that the amoeba (the host cell in this model) does not proliferate at 34°C in PYG medium. This choice of 34°C for subsequent co-culture experiments is unsuitable for studying a productive interaction, as the host is not totally viable. The authors must clarify this point.

12. Lines 255-301: This part is overly descriptive, detailing observational trends that are already visible in the figures. To improve clarity and impact, this section should be more synthetic, focusing on concisely stating the key findings and directing readers to the supporting data. A more effective strategy would be to structure the narrative around the key experimental variables. For instance, the 26°C condition led to a more sustained infection and higher host amoeba viability. The infection pattern in RPMI and PYG was similar.

13. Lines 302-306: I recommend moving this paragraph to the Discussion, since it provides an interpretation of the results

14. Lines 307-309: The interaction between *L. **amazonensis*** promastigotes and *Acanthamoeba* cysts is conceptually flawed and should be removed from the manuscript. Cysts are dormant, metabolically inactive stages characterized by a thick, protective cell wall. Its primary biological function is to resist environmental conditions, and no phagocytosis is expected.

15. Lines 310-314: Here, findings from a co-infection assay with amastigotes are mentioned, but this methodology is not described in the Materials and Methods section. Provide a complete description of the methodology for co-infection with amastigotes. Alternatively, as a preliminary, not a central, focus of the work, the authors should remove this mention and instead briefly comment on it in the Discussion as an informal observation.

16. Lines 321 – 325 (and corresponding methodology in Mat & Met), Figure 4: What is the rationale for the PCR assay? The authors present a more direct and functionally informative viability assay as definitive proof of parasite viability. In this context, the purpose of the conventional PCR is unclear and appears redundant. Also, PCR detection of *Leishmania* DNA does not confirm the presence of viable, replicating parasites, as the signal may originate from DNA released by degraded organisms.

17. Fig 6: The authors attribute membrane damage seen in a single SEM image to the interaction with *Leishmania*. However, a single micrography is not representative of a general pattern of interaction. Furthermore, the possibility that these structures are preparation artifacts cannot be ruled out.

Discussion

18. The Discussion begins with a summary of the methods and a repetition of basic findings. Consider removing this paragraph.

19. Line 374: This paragraph effectively cites previous studies with similar conclusions. This is an ideal point to highlight the novel contribution of the present work.

20. Winiecka-Krusnell et al. (2009) paper has a primary focus and title concern Toxoplasma, and the data on *L. tropica* appears to be a minor, comparative control. Mentioning that a similar pattern of clearance was incidentally observed by Winiecka-Krusnell et al. (2009) in a control experiment with *L. tropica* would prevent the reader from overestimating its role in the existing literature.

21. Line 417. Why would the lack of a three-dimensional structure be a limitation of the study?

22. Line 422: The statement that the objective was 'to evaluate whether *Acanthamoeba* could be used as a model organism sets an inappropriate expectation for the reader. A more precise aim would be: "to deeply characterize the fundamental interaction between *Acanthamoeba* and *L. **amazonensis***." Authors should consider reframing it throughout the manuscript.

23. Lines 451-464: The Conclusion currently repeats specific methodological details, results, and discussion points that have already been presented. It should be reframed to synthesize the main achievement.

## REVIEWER #2

The manuscript " Uptake and survival of *Leishmania **amazonensis*** in *Acanthamoeba **castellanii***: An adequate model organism?" by Geres et al. describes the *Acanthamoeba*-*Leishmania* interaction as a model to study how *Leishmania* is able to survive within a phagocytic cell and with other related approaches.

I have several comments as below:

- Throughout the manuscript the language is sometimes not specific, based on scientific "slang," or not completely accurate. It should be proofread to improve accuracy.

- Authors should focus on the virulence factors of *Leishmania*, such as the tolerance to the oxidative stress within macrophages or how *Leishmania* is able to survive within them. Does *Leishmania* disturb the phagosome maturation? What are the mechanisms behind this?

- The optimal temperature range for *Acanthamoeba* cultivation is 25-31ºC. At 34 and 37 ºC, were the trophozoites growing flattened onto coverslip surface, or were tending to become rounded? Assays were performed for 3, 24, 48 and 72h. This conditions stimulate the trophozoites-to-cysts transition, and rounded cells are going to gradually leak the ability of phagocytosis. *Acanthamoeba* is very sensitive to changes in nutrients depletion and cellular density. How many *Acanthamoeba* and *Leishmania* cells composed the inocula based on 1:10 and 1:20 ratios? I think that the first 12h of interaction were important to evaluate phagocytosis dynamics.

- How was cell viability calculated after mechanical lysis with needle passages? Were *Leishmania* and *Acanthamoeba* separately subjected to the same lysis protocol to compare cell viability? How can you infer that *Leishmania* was killed by *Acanthamoeba* and not by the mechanical lysis procedure?

- Why was the co-culture timepoint of 2h chosen for scanning electron microscopy assay? What were the criteria?

- How many trophozoites were counted per slide in relation to the percentage shown in Fig 1? Were only internalized cells considered, or were other events, such as interaction with surface cells, also taken into account? The events of surface interaction and internalized cells should be compared to assess the phagocytosis efficiency over time. This article may help you (https://doi.org/10.1016/j.micinf.2023.105153)

- Please, explain the reason for the trophozoites augmentation (virtually no cysts) at 37 ºC (Fig. 1S), considering that the optimal temperature range for *A. **castellanii*** (ATCC 30010) cultivation is 25-31 ºC.

- Increase the size of Figs 1, 2, 3, 5, 1S, 2S

- The meaning of the term "intact trophozoites" is unclear, especially in the context of line 268. Please clarify its meaning or replace it throughout the manuscript.

- Line 278, "In addition, a very limited number of trophozoites were heavily parasitized with *L. **amazonensis*** (Figure 2J-L)." This may have occurred due to the prolonged co-culture period (> 6 h). Note the presence of a rounded trophozoite in Fig. 2L, probably in the process of becoming a cyst. Therefore, this cell is no longer expected to have phagocytic ability. Please provide the number of trophozoites interacting with *Leishmania* per slide and associate this information with the percentage for each time point of the co-culture. For example, one hundred or more trophozoites per slide can provide valuable information about the interaction between *Leishmania* and *Acanthamoeba* (cell surface interaction versus internalized cells).

- Lines 307-312, It is a long-established that cysts are a non-phagocytic, protective, and dormant stage in the parasite's life cycle. It was expected that this would happen.

- Lines 376-380, there is evidence that *Acanthamoeba* possesses a surface receptor similar to the TLR-5 of mammalian phagocytic cells, which recognizes flagellin. This article may be useful (https://doi.org/10.1099/mic.0.001386).

- Line 400, write "over time" instead of "over tine". I recommend that the author submit the manuscript to an English editing service.

- Lines 404-406, "only cytokine-activated macrophages were able to inhibit *Leishmania* growth (Bogdan 2020, Santos et al. 2024). Could it be write this way?

- Line 409, please, write "oxidative stress" instead of "oxidative attack".

- Lines 414-416, please, provide details about the method for NO measurement.

- Line 427, should delve deeper into the discussion "different strategies to kill microorganisms". It appears that a similar apoptosis pathway exists for protozoan parasites. If *Acanthamoeba* dies, should *Leishmania* also die (inside the amoeba)?. This article (https://doi.org/10.1186/1756-3305-4-44) may help you find recent studies based on this topic.

- Lines 440-448, you should explore the hypothesis that *Acanthamoeba* and *Leishmania* can interact within mosquitoes.

- Lines 431-438, please, clarify the discussion about *Acanthamoeba* in relation to the "trojan horse of the microbial world", "cystic resistance" and "microbial biofilm" terms.

Please, present a conclusion without rewriting your results. What gaps were filled? What was your contribution to the scientific knowledge about the *Acanthamoeba*-*Leishmania* interaction? What is possible to do and what should future studies focus on?

## AUTHORS' RESPONSE TO THE REVIEWERS

REVIEWER COMMENTS: Reviewer:

1. This manuscript investigates the use of *Acanthamoeba* as a model to study *Leishmania **amazonensis*** infection. [...] Considering this established context, I have three overall considerations of the manuscript: 1) It is crucial to define its novel contribution to avoid the perception of merely confirming or extending prior findings. [...] We agree with the reviewer and appreciate this important point. We have revised the manuscript to more explicitly define its novel contributions. 2) The stated objective of evaluating *A. **castellanii*** as a model organism seems somewhat broad [...] We agree with the reviewer's assessment. Accordingly, we have revised the main objective of the study to focus on understanding the biology of the interaction between *Acanthamoeba* and *Leishmania* [...]. 3) The manuscript needs a clearer logical story. [...] We thank the reviewer for this important feedback. We have revised the manuscript to improve its overall logical flow [...]. In doing that, we decided to remove the PCR assay which did not directly contribute to the central objective of the study.

Abstract: 1. Lines 28-31: [...] We thank the reviewer for this valuable suggestion. The abstract was revised, and the opening sentences were restructured to be more direct and focused on the research challenge, as recommended (Line 27-31).

2. Line 33: [...] We thank the reviewer for this comment. The objective was revised to better align with the experimental approach employed in this study (Line 32-34).

3. Line 45: [...] We thank the reviewer for this valuable suggestion. The conclusion was revised. The term "not relevant" was removed and the text was reframed to emphasize the model's specific suitability (Line 44-45).

4. Lines 31,33,35,44: Revise the binomial designation *A. castelllanii*, considering the comment #5 below. We thank the reviewer for this important taxonomic recommendation. We revised the manuscript to improve taxonomic accuracy and readability as suggested. In response, we have adopted the genus-level nomenclature (*Acanthamoeba*) throughout the manuscript to avoid taxonomic ambiguity. [...] We also adapted the article title to reflect the new chosen nomenclature.

5. Introduction. [...] We thank the reviewer for this important taxonomic recommendation. We revised the manuscript to improve taxonomic accuracy and readability, as recommended by the point 4 made above (e.g., lines 38, 43, 88, 92, 96).

6. Line 101: [...] We thank the reviewer for this important suggestion. The mention of Yaravirus has now been supported by the suggested citations (Lines 99-100).

7. Lines 118-124: [...] We thank the reviewer for this important suggestion. We have reformulated this section to more clearly emphasize the core objective of the study, and the methodological details have been relocated to the Materials and Methods section (Lines 117-118).

8. Material and Methods. Line 135: [...] We thank the reviewer for this important taxonomic recommendation. We revised the manuscript to improve taxonomic accuracy and readability, as answered in the point 4 (Lines 134 – 137).

9. The standard medium [...] Please remove the 'W' [...]. We thank the reviewer for pointing this out. The manuscript was thoroughly revised to ensure consistent use of the conventional nomenclature PYG (Peptone–Yeast Extract–Glucose), and the letter "W" was removed throughout the text accordingly.

10. Line 179-182: [...] We thank the reviewer for this helpful comment. The text was revised to improve clarity and flow (Line 181). In addition, we included a specific reference related to amoeba viability assessment (https://doi.org/10.1371/journal.pntd.0011878) (Line 182).

11. Results. Line 250-255 and Fig S1: [...] We thank the reviewer for this detailed and constructive comment. [...] Regarding the choice of temperature, we agree that *Acanthamoeba* does not proliferate at 34 °C in PYG medium, as shown in Figure S1F. However, although proliferation is impaired, the amoebae remain viable for up to 72h, predominantly in the trophozoite stage, and subsequently enter an early encystment process. [...] We have modified the text to include this explanation (Lines 243-249).

12. Lines 255-301: [...] We agree with the reviewer that this section was overly descriptive. Accordingly, we have revised the text to make it more concise and synthetic [...] (Lines 250-272).

13. Lines 302-306: [...] We thank the reviewer for pointing this out. We have replaced this paragraph to the Discussion section (Lines 339-342).

14. Lines 307-309: [...] We thank the reviewer for this comment and agree with the assessment. This paragraph was removed from the manuscript.

15. Lines 310-314: [...] We thank the reviewer for this comment and agree with the assessment. We have removed this mention.

16. Lines 321 – 325 [...] Figure 4: What is the rationale for the PCR assay? [...] We agree with the reviewer's assessment. Therefore, we have removed this assay and its corresponding methodology from the manuscript.

17. Fig 6: [...] We thank the reviewer for this comment; however, the membrane alterations were consistently observed across multiple SEM fields, indicating a recurrent pattern rather than an isolated event. Importantly, a control sample consisting of only amoebae processed in parallel under identical preparation conditions, but without *Leishmania* interaction, did not display these alterations. This strongly suggests that the observed structures are not preparation artifacts.

18. Discussion. The Discussion begins with a summary of the methods [...] We agree with the reviewer's assessment. Therefore, we have removed this paragraph.

19. Line 374: [...] We thank the reviewer for this comment. We have reformulated this paragraph highlighting the novel contribution of our work (Lines 325 – 338).

20. Winiecka-Krusnell et al. (2009) [...] We fully agree with the reviewer's observation and have revised the text to explicitly indicate that this finding represents an incidental observation within the cited study (Lines 365 – 367).

21. Line 417. Why would the lack of a three-dimensional structure be a limitation of the study? We thank the reviewer for raising this point. This paragraph was misplaced in the Discussion. Accordingly, we have relocated it to the beginning of the Discussion section [...] (Lines 325 – 338).

22. Line 422: [...] We thank the reviewer for this insightful comment and agree [...]. Accordingly, we have revised the manuscript to clarify that the central objective of the study is to provide an in-depth characterization of this host–parasite interaction.

23. Lines 451-464: [...] We thank the reviewer for this comment. We have revised the Conclusion section to improve its clarity and focus [...] (Lines 423-430).

Reviewer: 2

- Throughout the manuscript the language is sometimes not specific [...] Thank you for your valuable feedback regarding the language in our manuscript. We have carefully revised the entire text. Authors should focus on the virulence factors of *Leishmania* [...] Thank you for these excellent and insightful suggestions. [...] Therefore, the primary scope and contribution of our current work was to establish a more accurate morphological analysis and a detailed quantitative kinetic profile of this host-parasite interaction [...].

- The optimal temperature range for *Acanthamoeba* cultivation is 25-31ºC [...] We thank the reviewer for these insightful questions [...] We acknowledge that the optimal proliferation range for *Acanthamoeba* is 25-31°C. The use of 34°C was an experimental choice, despite it not being a standard growth condition. Our rationale was based on the biological relevance of 34°C as the skin temperature of mammalian hosts [...]. Regarding the inoculum concentrations for the 1:10 and 1:20 ratios: For the co-culture assays, 3 × 10^5^ trophozoites were plated. To achieve the stated ratios: For a 1:10 ratio (amoeba:promastigote), 3 × 10^6^ promastigotes were added. For a 1:20 ratio, 6 × 10^6^ promastigotes were added. [...] This information were added in the material and Methods section [...] (Lines 154-157).

- How was cell viability calculated after mechanical lysis with needle passages? [...] Thank you for raising these important methodological points [...] Cell viability of the recovered *Leishmania* promastigotes was assessed based on the capacity for subsequent proliferation in culture. [...] To specifically rule out that the mechanical lysis procedure itself was responsible for reduced parasite viability, we performed a critical control experiment. Free-living *Leishmania* promastigotes (not exposed to amoebae) were subjected to the exact same mechanical lysis protocol [...]. Our conclusion that *Leishmania* was killed by *Acanthamoeba* and not by the experimental procedure is supported by a convergence of evidence [...].

- Why was the co-culture timepoint of 2h chosen for scanning electron microscopy assay? [...] Thank you for the opportunity to clarify this methodological point. The 2-hour co-culture timepoint for scanning electron microscopy (SEM) was selected based on direct evidence from our preliminary kinetic analyses. [...] This specific timepoint represents a critical phase in the interaction where a significant number of *Leishmania* promastigotes are adhered to the surface of *Acanthamoeba* trophozoites but have not yet been fully internalized.

- How many trophozoites were counted per slide in relation to the percentage shown in Fig 1? [...] Thank you for these pertinent questions regarding the quantitative analysis. To determine the percentages of trophozoites with *L. **amazonensis***, 200 amoebas were counted per coverslip [...] (Line 166). [...] We acknowledge that distinguishing between adhesion and true internalization is crucial for precise phagocytosis efficiency metrics [...].

- Please, explain the reason for the trophozoites augmentation (virtually no cysts) at 37 ºC (Fig. 1S) [...] Thank you for this insightful question [...] We acknowledge that the optimal proliferation range for *Acanthamoeba* is 25-31°C. The use of 34°C was an experimental choice [...] We have revised the text to make this rationale clearer and to avoid any misinterpretation (Lines 243-249).

- Increase the size of Figs 1, 2, 3, 5, 1S, 2S. Thank you for your comment, as suggested, we have increased the size and resolution of figures 1, 2, 3, 5, 1S, and 2S [...].

- The meaning of the term "intact trophozoites" is unclear [...] We thank the reviewer for raising this important point. We have revised the manuscript and replaced the term "intact trophozoites" by viable amoeba throughout the manuscript.

- Line 278, "In addition, a very limited number of trophozoites were heavily parasitized with *L. **amazonensis*** (Figure 2J-L)." [...] We thank the reviewer for the observation. [...] The highly parasitized trophozoites shown in Figures 2J-L were captured at the 3-hour time point, not after a prolonged co-culture (>6h). This indicates that intense parasite association can occur even during the early phases of interaction. [...]

- Lines 307-312, It is a long-established that cysts are a non-phagocytic, protective, and dormant stage [...] We thank the reviewer for this comment and agree with the assessment. As the observations regarding the interaction between *L. **amazonensis*** promastigotes and *Acanthamoeba* cysts do not provide novel insight to the current literature, this paragraph was removed from the manuscript.

- Lines 376-380, there is evidence that *Acanthamoeba* possesses a surface receptor similar to the TLR-5 of mammalian phagocytic cells [...] We thank the reviewer for this valuable suggestion. The information highlighted in the cited article has now been incorporated into the Discussion section [...] (Lines 345-348).

- Line 400, write "over time" instead of "over tine" [...] We thank the reviewer for pointing out this typographical error. The term "over tine" has been corrected to "over time" (Line 369).

- Lines 404-406 [...] This information was misplaced in the Discussion, and could compromise clarity and understanding of the text, so it was removed from the discussion.

- Line 409, please, write "oxidative stress" instead of "oxidative attack". We thank the reviewer for this suggestion. The term "oxidative attack" has been replaced with "oxidative stress" (Line 378).

- Lines 414-416, please, provide details about the method for NO measurement. We thank the reviewer for this comment. [...] we have added a brief and informal methodological description of the NO measurement using the Griess method in this same section, and referenced (Line 381).

- Line 427, should delve deeper into the discussion "different strategies to kill microorganisms" [...] We appreciate this insightful comment and the suggested reference. In our experimental conditions, while *Leishmania* was all cleared, the *Acanthamoeba* trophozoites largely remained intact, suggesting that parasite death may not be linked to a classical apoptosis-like pathway shared by both organisms. [...]

- Lines 440-448, you should explore the hypothesis that *Acanthamoeba* and *Leishmania* can interact within mosquitoes. We thank the reviewer for this valuable suggestion. We have revised and reformulated this section.

- Lines 431-438, please, clarify the discussion about *Acanthamoeba* in relation to the "trojan horse of the microbial world", "cystic resistance" and "microbial biofilm" terms. We thank the reviewer for pointing out this issue. We have clarified and refined the discussion [...] (Line 397 – 404).

Please, present a conclusion without rewriting your results [...] We thank the reviewer for this important comment. We have substantially revised the Conclusion (Lines 423 – 430).

# Reviewer report: REVIEWERS' COMMENTS

## REVIEWER #1

The authors have been responsive to reviewer comments and modifications have greatly improved rigor and readability of the manuscript.

My two suggestions for the manuscript are:

Lines 96-100. I think the amoeba-fungus interaction has been explored more thoroughly with *Cryptococcus*, at least in the last two decades, than with other fungi, such as dermatophytes. Nothing against mentioning new findings, but I think more established approaches to amoeba-fungus interaction should also be cited in this context (https://doi.org/10.1016/j.micinf.2023.105153).

Lines 389-401. I suggest mentioning, in addition to the bacteria in this paragraph, that *Acanthamoeba* is capable of modulating (increasing) the virulence of *Cryptococcus* (https://doi.org/10.1016/j.micinf.2023.105153), an important human pathogenic fungus that causes more than 600,000 deaths per year worldwide. This is important to show the diversity of microorganisms of medical interest that the amoeba can interact with and influence their virulence from the environment, as well as to reinforce the importance of using *Acanthamoeba* as a tool for cell interaction studies.

## REVIEWER #2

The revisions are satisfactory and address all recommended points. Nevertheless, the added text would benefit from a final language polish. I would recommend a language edit to improve flow and conciseness in the following specific parts of the text:

1) Lines 365-367 (Original: "…who incidentally observed a similar pattern in a control experiment with *L. tropica*, which showed a decrease in *Leishmania* internalized by the amoeba, which were completely cleared after 48h of co-culture.). Suggestion: "…who incidentally observed a similar pattern in a control experiment with *L. tropica*. In their study, internalized *Leishmania* decreased and were completely cleared from the amoeba after 48h of co-culture."

2) Lines 385-388 (Original: "As a new contribution to literature, we have determined that the optimal experimental condition was RPMI medium at 26°C, by live microscopy we have seen that *L. **amazonensis*** is briefly internalized by *Acanthamoeba*, however, its survival rate decreased after 24 h."). Suggestion: "As a new contribution to the literature, we determined that the optimal experimental condition was RPMI medium at 26°C. Live microscopy revealed that *L. **amazonensis*** is briefly internalized by *Acanthamoeba*; however, its survival rate decreased after 24 h."

3) Lines 398-401 (Original: "While amoebae are able to eliminate some microorganisms from the environment, they can also internalize pathogens such as *Legionella* and *Mycobacterium* into their vacuoles, protecting them and enhancing their virulence [36].") Suggestion: "While amoebae can eliminate some microorganisms, they also internalize pathogens like *Legionella* and *Mycobacterium* into their vacuoles, thereby protecting these bacteria and enhancing their virulence [36]."

4) Lines 401-404: (Original: "Amoeba when challenged, transforms into double-walled cysts, structures remarkably resilient. This cystic resistance ensures the environmental persistence of both the protozoan and intracellular pathogens. It can be widespread in practically any environment [12,13,16]."). Suggestion: "When challenged, the amoeba transforms into a remarkably resilient, double-walled cyst. This resistance ensures the environmental persistence of both the protozoan and any intracellular pathogens, allowing *Acanthamoeba* to thrive in virtually any environment."

EDITOR COMMENTS:

I kindly ask the authors to incorporate the minor adjustments suggested by the reviewers and to take this opportunity to perform a comprehensive revision of the English language and grammar with an expert.

## AUTHORS' RESPONSE TO THE REVIEWERS

REVIEWER COMMENTS:

Reviewer: 1

Lines 96-100. I think the amoeba-fungus interaction has been explored more thoroughly with *Cryptococcus* [...] Thank you for the valuable suggestion. The recommended article has now been cited and appropriately incorporated into the revised manuscript (Lines 107, 110). We also took this opportunity to carefully revise the references and improve the English language.

Lines 389-401. I suggest mentioning, in addition to the bacteria in this paragraph, that *Acanthamoeba* is capable of modulating (increasing) the virulence of *Cryptococcus* [...] Thank you for the suggestion. The recommended reference has been incorporated into the revised manuscript (Lines 384-388) to highlight the interaction between *Acanthamoeba* and *Cryptococcus*.

Reviewer: 2

The revisions are satisfactory and address all recommended points. Nevertheless, the added text would benefit from a final language polish [...] Thank you for your careful evaluation and constructive recommendation. We have performed a comprehensive revision of the entire manuscript to further improve the English language, grammar, clarity, and overall readability. The manuscript has also undergone professional English language editing by Editage prior to its submission to MIOC. A certificate confirming this linguistic review is available and has been attached.

1) Lines 365-367 [...] Thank you for the suggestion. The text has been revised accordingly to improve clarity and readability (Lines 351 – 353).

2) Lines 385-388 [...] Thank you for the suggestion. The text has been revised accordingly to improve clarity and readability (Lines 371 – 374).

3) Lines 398-401 [...] Thank you for the suggestion. The text has been revised accordingly to improve clarity and readability (Lines 385-388).

4) Lines 401-404 [...] Thank you for the suggestion. The text has been revised accordingly to improve clarity and readability (Lines 388-391).

EDITOR COMMENTS:

I kindly ask the authors to incorporate the minor adjustments suggested by the reviewers and to take this opportunity to perform a comprehensive revision of the English language and grammar with an expert.

Thank you for your careful evaluation and constructive recommendation. We have performed a comprehensive revision of the entire manuscript to further improve the English language, grammar, clarity, and overall readability. The manuscript has also undergone professional English language editing by Editage prior to its submission to MIOC. A certificate confirming this linguistic review is available and has been attached.

# Reviewer report: REVIEWERS' COMMENTS

## REVIEWER #1

The revisions are satisfactory and address the recommended points.

## REVIEWER #2

English editing and formatting are required, as recommended by the editor. The manuscript has been improved and now meets the requirements for acceptance by MIOC.
